# A SCOPING REVIEW OF PHYSICAL ACTIVITY IN ADULTS OVER 50 WITH AUTISM: MANY BARRIERS AND SPARSE DATA

**DOI:** 10.1093/geroni/igad104.3150

**Published:** 2023-12-21

**Authors:** Connie Kartoz, Rebeca Jefferson, Antigone Antonakakis, Chase Eisenberg

**Affiliations:** The College of New Jersey, Ewing, New Jersey, United States; The College of New Jersey, Ewing, New Jersey, United States; The College of New Jersey, Ewing, New Jersey, United States; The College of New Jersey, Ewing, New Jersey, United States

## Abstract

The population of older adults with autism is growing, yet the literature for this group is still relatively sparse (Mason et al., 2022). Adults with autism are reported to have multiple co-morbidities and a reduced life span (Hand et al., 2020). While physical activity (PA) is known to reduce chronic disease burden, extant research indicates young adults with autism do not engage in physical activity (PA) with the same frequency as neurotypical peers. Little is known about PA for older adults with autism. This scoping review aimed to explore research about PA for older adults with autism. Following the PRISMA-ScR guidelines, 5 databases were searched (2018 and later) with keywords ‘autism’, ‘older adults’, ‘PA’ and age group delimiters. Initially, no articles were found, and the search was expanded to include all adults. After eliminating duplicates, 121 studies were reviewed, with 31 meeting the criteria (PA, autism and no participants less than 18) and 9 having any participants over 50; in each of these 9 studies, the number of participants over 50 was less than 100. Only 2 studies focused on adults over 50. Level of evidence was low. The limited data suggests those aging with autism do not engage in recommended levels of PA, experience barriers to the same, but that interventions improve PA and possibly quality of life. Based on this review, there is a dire need to design interventional research regarding increasing PA in older adults with autism to improve equitable health outcomes for this at-risk group.

